# Publisher’s Note added on 24 January 2014

**DOI:** 10.3390/cells1040951

**Published:** 2014-01-24

**Authors:** Shu-Kin Lin

**Affiliations:** MDPI, Klybeckstrasse 64, Postfach, CH-4057 Basel, Switzerland; E-mail: lin@mdpi.com

Publisher’s Note added on 24 January 2014: *Cells*, Volume 1, Pages 951–960 are taken as blank pages because of a pagination error in the preceding paper. We apologize for any inconvenience caused to our readers.

